# EDL-VINS: Protocol-Controlled Evaluation of an EDLines and Line-Flow Point–Line Visual–Inertial System

**DOI:** 10.3390/s26175508

**Published:** 2026-08-30

**Authors:** Bo Yang, Weixian Li, Xin Han, Junlin Deng, Xueshan Gao, Ning Wu

**Affiliations:** Key Laboratory of Beibu Gulf Offshore Engineering Equipment and Technology, Beibu Gulf University, 12 Binhai Road, Qinnan District, Qinzhou 535011, China; 2402010122@stu.bbgu.edu.cn (B.Y.); liweixian9016@163.com (W.L.); 15871789630@whut.edu.cn (X.H.); xueshan.gao@bit.edu.cn (X.G.)

**Keywords:** visual–inertial localization, point–line fusion, line optical flow, EDLines, trajectory evaluation, simultaneous localization and mapping, mobile robot navigation

## Abstract

Comparisons of point–line visual–inertial systems can be distorted when trajectories cover different time intervals or when metric estimates receive scale correction. This study asks whether the recorded performance of an EDLines and line-flow system persists under identical temporal support and metric-preserving alignment. The evaluated EDLines (Edge Drawing Lines) visual–inertial navigation system (EDL-VINS) combines conditioned line extraction, pyramidal line-flow association, and Plücker-line factors within a point–line–inertial sliding window. To answer this question, we audit the retained trajectories across all eleven EuRoC sequences, resample the three systems onto a common time grid, and evaluate them without scale correction. The robustness of the resulting comparison is then tested under alternative alignment and timestamp support; gap-aware interpolation; and shorter-horizon, rotational, and archived-output variation analyses. EDL-VINS retains the lowest aggregate translational and rotational trajectory error, although sequence-specific exceptions and short-horizon results show that the ordering is not universal. These findings provide an auditable characterization of the evaluated configuration and show why temporal support, alignment, and data provenance must be explicit in visual–inertial comparisons.

## 1. Introduction

Visual–inertial localization is a foundational capability for autonomous mobile robots operating in environments where global navigation satellite system signals are unavailable or unreliable. Microaerial vehicles inspecting industrial facilities, ground robots navigating warehouses, and handheld devices mapping indoor spaces all depend on accurate, real-time motion estimation from onboard cameras and inertial sensors. In these scenarios, structural features such as walls, corridors, and door frames provide rich line information that complements point features, particularly when texture is sparse or illumination changes rapidly. The ability to reliably exploit these line features while maintaining computational efficiency is therefore directly relevant to the practical deployment of autonomous systems in built environments.

Visual–inertial localization combines camera images with inertial measurements to estimate metric motion when external positioning is unavailable. Optimization-based systems such as VINS-Mono and OKVIS have established accurate point-based formulations with inertial measurement unit (IMU) pre-integration and bounded-window estimation [1,2]. Their visual support can nevertheless weaken around blank walls, repeated structures, motion blur, and abrupt illumination changes. Point–line systems address this problem by adding structural observations that remain detectable where localized corner texture is sparse [3,4]. However, the value of these additions is difficult to judge when compared trajectories do not share the same temporal support or alignment model.

Within point–line systems, the benefit of line features is coupled to their computational cost. Descriptor-based pipelines repeatedly detect, describe, and match segments, whereas Edge Drawing Lines (EDLines) and line-flow approaches reduce this burden through edge drawing and temporal propagation [5,6]. The resulting system is not defined by a detector alone: finite image segments must be associated across frames, converted into spatial line landmarks, and combined with point and inertial factors. Consequently, a credible engineering claim must connect the complete configuration to trajectory evidence while keeping detector-stage timing separate from end-to-end performance.

Beyond front-end design, trajectory comparison introduces a second problem. Different implementations may produce poses at different rates, start or end at different times, and be evaluated with either the special Euclidean group SE(3) for rigid alignment or the similarity group Sim(3) for scale-corrected alignment. Scale correction can conceal metric scale error even though a visual–inertial system is expected to recover scale. Evaluating each method on its native timestamps can also assign different temporal support to nominally paired results. These choices can change both error magnitudes and method rankings, yet they are often condensed into one absolute trajectory error (ATE) table.

Accordingly, this study focuses on whether the recorded performance pattern of an EDLines and line-flow point–line–inertial system persists when all methods are compared on the same temporal support without scale correction. EDL-VINS denotes the evaluated implementation, which integrates EDLines conditioning, descriptor-free line flow, and geometric line factors in a VINS-Mono-derived estimator. The study contributes a documented system description and a protocol-controlled characterization of this implementation.

The novelty of this study lies not in proposing new algorithmic components, but in establishing an evaluation methodology that isolates protocol artifacts from estimator performance. Prior comparisons of point–line visual–inertial systems have typically reported results under varying temporal support, inconsistent alignment models, and unacknowledged scale corrections, making it difficult to determine whether observed performance differences reflect genuine estimator improvements or evaluation artifacts. By enforcing identical temporal support, metric-preserving alignment, and transparent sensitivity analysis, this work provides the first auditable characterization of an EDLines/line-flow configuration that separates genuine capability from protocol-induced variation.

The paper makes three contributions:

1. It provides a coherent specification of the evaluated EDL-VINS processing chain, from EDLines candidate conditioning and pyramidal line flow to multi-view Plücker-line initialization and joint point–line–IMU optimization.
2. It establishes an auditable trajectory protocol for all eleven EuRoC sequences: the three methods are evaluated on a shared 5 Hz time grid with SE(3) alignment, while interpolation-gap, native-support, and Sim(3) results test the stability of the comparison.
3. It releases the complete set of trajectory products, archived evaluation arrays, integrity hashes, and analysis code and uses them to characterize aggregate accuracy, fixed-lag displacement behavior, alignment sensitivity, sequence-specific exceptions, and archived-output variation on two sequences.

The remainder of the paper first positions the system among point–line visual–inertial methods, then defines the implementation and evaluation protocol, and finally interprets the engineering significance of the recovered evidence.

## 2. Related Work

### 2.1. Point–Line Visual–Inertial Estimation

Geometric line landmarks have been incorporated into both visual simultaneous localization and mapping (SLAM) and visual–inertial odometry (VIO). PL-SLAM combines point and line observations in a stereo formulation [7]. PL-VIO represents spatial lines with Plücker coordinates and a minimal orthonormal parameterization, allowing point, line, and inertial factors to be optimized jointly [3]. PL-VINS extends the VINS-Mono family with line observations and a modified line front end [4], while PLI-VINS adapts extraction and segment management for indoor environments [8]. These systems establish the estimator-side role of lines but retain nontrivial front-end cost.

More recent systems improve robustness through lightweight tracking or additional structural constraints. LRPL-VIO uses IMU-predicted photometric tracking of line samples before a filter update [9]. PLE-SLAM extends rotation–translation-decoupled initialization to point–line features with efficient IMU bias estimation [10], while PKL-VINS strengthens EDLines processing through dynamic length thresholds and keypoint-guided extension [11]. Adaptive feature strategies have also been explored, for example, through vanishing-point screening of line observations and plane-normal geometric constraints [12]. Structural-line constraints and adaptive point–line weighting have also been used to strengthen estimation under challenging motion or texture [13,14]. Learned line features offer another route under illumination change but require a different computational budget and training dependency [15]. Stepwise marginalization has also been applied to point–line fusion to reduce sliding-window coupling while preserving line constraints [16].

Recent systems continue to refine the same point–line recipe along the front-end, association, and estimator interfaces. POL-VIO [17] and IPL-VI-SLAM [18] strengthen line-feature processing and loop closure, OKVIS2-X [19] generalizes the estimator into a configurable multi-sensor system, and LaMAria [20] exposes the robustness gap that remains at city scale. None of them, however, makes the temporal-support and alignment choices auditable—precisely the gap this study targets.

### 2.2. Efficient Line Detection and Association

The Line Segment Detector (LSD) forms line-support regions from image gradients [21], and the Line Band Descriptor (LBD) supports appearance-based segment matching [22]. EDLines instead traces edge chains and validates fitted segments, providing a lower-cost detector in many image regimes [5]. Recent applications have combined EDLines with Plücker coordinate parameterization and length-based filtering for point–line optimization in domain-specific deployments [23,24]. EPLF-VINS combines short-segment fusion, spatial distribution control, adaptive extraction, and line optical flow with an endpoint-independent spatial-line measurement [6]. EPL-VINS subsequently combines optimized EDLines processing and line flow with an adaptive estimator residual [25]. EDL-VINS belongs to this family and uses the same central distinction: finite image support is propagated temporally, whereas persistent estimator geometry is carried by a spatial line.

Table 1 positions the evaluated configuration at the detector–association–estimator interfaces and clarifies how its processing chain relates to representative point–line visual–inertial systems.

In particular, the present contribution differs from EPL-VINS’s adaptive-residual formulation: it evaluates the EDLines/line-flow configuration through a controlled and auditable trajectory protocol.

### 2.3. Trajectory Evaluation

Trajectory evaluation requires explicit choices about time association, alignment, and error metrics [26]. ATE summarizes global position disagreement after trajectory association and alignment, and the evo toolkit provides widely used implementations for odometry and SLAM evaluation [27]. Nevertheless, the selected options remain part of the experimental protocol. Scale correction is appropriate for scale-ambiguous monocular vision; for metric VIO, SE(3) alignment is the more stringent primary choice. Common temporal support is equally important when estimators emit poses at different rates or contain internal gaps. However, prior comparisons do not always show whether method rankings remain stable under common timestamp support and metric-preserving alignment. The present study addresses this evaluation gap by making both choices explicit and treating alternative protocols as sensitivity analyses rather than interchangeable measurements.

## 3. Materials and Methods

### 3.1. System Architecture

**Figure 1:**
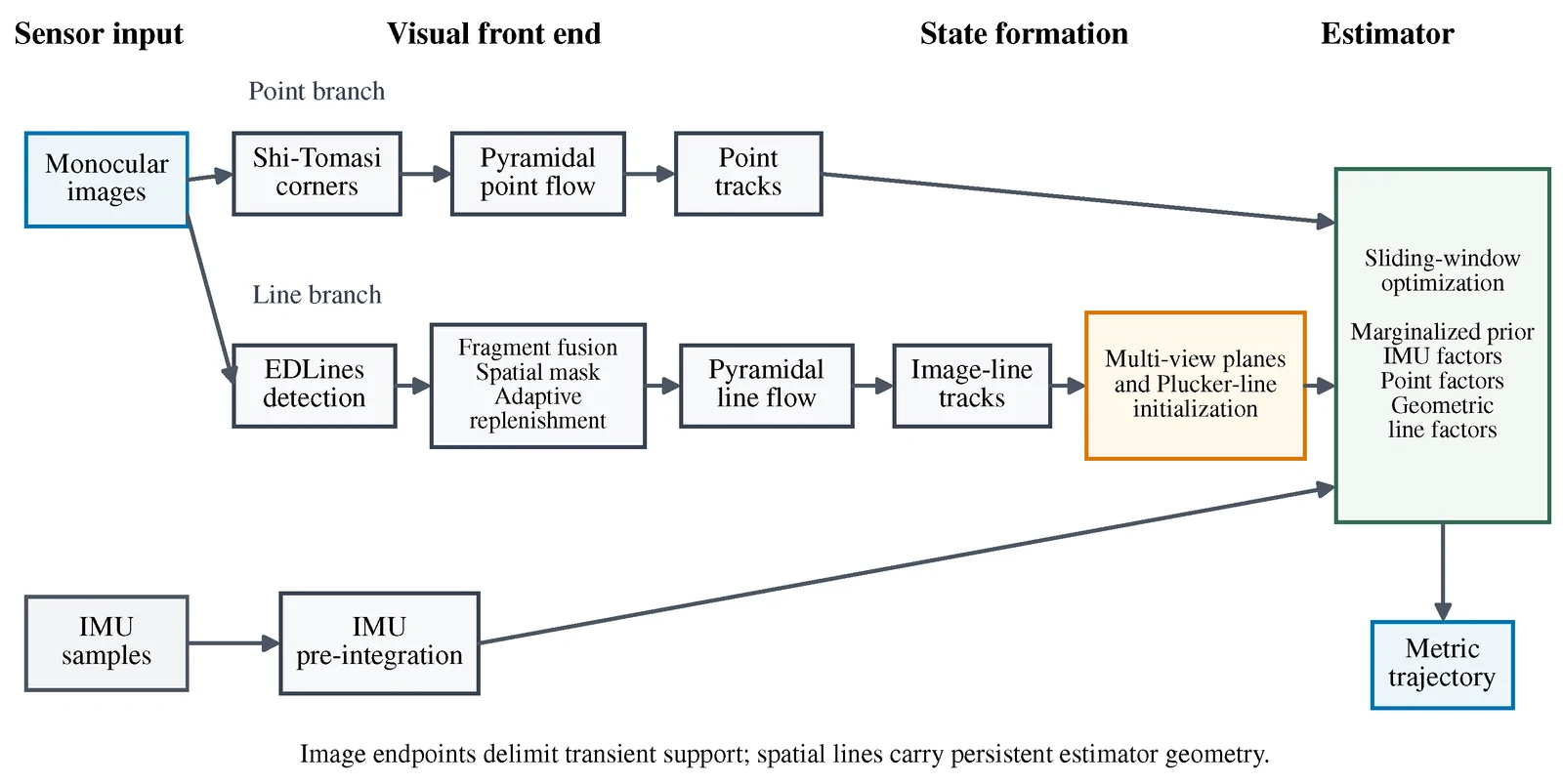
EDL-VINS processing chain. Finite image endpoints delimit the support used by temporal line association. Accepted image-line tracks initialize persistent spatial lines, whose geometric residuals enter the point–line–inertial sliding-window estimator. IMU denotes inertial measurement unit.

EDL-VINS follows a two-rate visual–inertial architecture. Camera images supply point and line observations, and higher-rate inertial measurements are pre-integrated between keyframes. The point branch uses Shi–Tomasi corners and pyramidal Lucas–Kanade tracking [28]. The line branch applies EDLines-based detection and candidate conditioning and then propagates the resulting image-line support through a pyramidal line-flow model. Multi-view image lines initialize spatial landmarks, and a fixed-size sliding window jointly optimizes navigation states, point landmarks, line landmarks, and a marginalized prior. Figure 1 shows this chain.

The implementation uses algorithmic elements established by EPLF-VINS and PL-VINS [4,6]. Section 3.2, Section 3.3 and Section 3.4 document how these elements are instantiated in the evaluated system. The complete processing chain is then treated as the unit of analysis and linked to the trajectory evidence under the controlled protocol.

### 3.2. EDLines Candidate Conditioning

Let $u_{s}={\left[u_{s},v_{s}\right]}^{T}$ and $u_{e}={\left[u_{e},v_{e}\right]}^{T}$ be the finite endpoints of an image segment. Its unoriented angle θ∈[0,π) and pixel length *L* are

(1) $$\theta =\mathrm{mod}\;\left(\mathrm{atan}2(v_{e}-v_{s},u_{e}-u_{s}),\pi \right),\;L={∥u_{e}-u_{s}∥}_{2}.$$

For two candidates *m* and *n*, define their unoriented angular difference $\Delta \theta_{mn}$ and minimum endpoint separation $d_{mn}$ as

(2) $$\begin{array}{cc} \Delta \theta_{mn} & =\min\;\left(|\theta_{m}-\theta_{n}\left|,\pi -\right|\theta_{m}-\theta_{n}|\right),\\ d_{mn} & =\min_{\alpha ,\beta \in \{s,e\}}{∥u_{m}^{\alpha }-u_{n}^{\beta }∥}_{2}. \end{array}$$

Nearby, similarly oriented fragments are merged to reduce duplicated support. A binary image mask suppresses insertion of additional segments around already accepted candidates, improving spatial coverage. When the active set falls below the 40-segment trigger count, the front end recovers structural cues by lowering the EDL+ gradient threshold and re-running the EDLines detector, discarding segments shorter than 30 px and capping the recovered set at a per-frame budget of 100 segments before it is passed to the estimator. Table 2 lists the numerical values preserved in the implementation record.

### 3.3. Pyramidal Line-Flow Association

Line flow follows the brightness-constancy and collinearity model used in EPLF-VINS [6]. Consider *M* sampled pixels on a segment. With the first endpoint as origin, sample *n* at signed along-line distance $\lambda_{n}$ has coordinate

(3) $$q_{n}=\left[\begin{array}{c} u_{n}\\ v_{n} \end{array}\right]=\left[\begin{array}{c} u_{s}+\lambda_{n}\cos\theta \\ v_{s}+\lambda_{n}\sin\theta \end{array}\right].$$

Let $g={\left[g_{u},g_{v},g_{\theta }\right]}^{T}$ contain image-plane translation and a small in-plane rotation between consecutive frames. First-order linearization gives the sample displacement

(4) $$\Delta q_{n}\left(g\right)=\left[\begin{array}{c} g_{u}-\lambda_{n}\sin\theta \;g_{\theta }\\ g_{v}+\lambda_{n}\cos\theta \;g_{\theta } \end{array}\right].$$

For image intensities $I_{i}$ and $I_{j}$ in adjacent frames *i* and *j*, respectively, the photometric residual is

(5) $$r_{n}\left(g\right)=I_{j}\;\left(q_{n}+\Delta q_{n}\left(g\right)\right)-I_{i}\left(q_{n}\right).$$

The motion increment minimizes $\sum_{n=1}^{M}r_{n}{\left(g\right)}^{2}$ by Gauss–Newton iteration. A coarse-to-fine image pyramid supplies the estimate at the next finer level, allowing the same three-parameter model to accommodate larger inter-frame motion. When the number of active line tracks falls below the trigger in Table 2, EDLines replenishes the set instead of performing an all-pairs descriptor search.

The line-flow implementation tracks segment endpoints through a 21×21-pixel search window across three pyramid levels. Tracked endpoints that fall outside the image border after propagation are rejected. The point-feature branch uses the same window and pyramid configuration with a forward–reverse consistency check: a point is retained only if the reverse-tracking displacement does not exceed 0.5 pixels. These settings, together with the numerical values in Table 2, constitute the parameter set preserved in the evaluated configuration.

### 3.4. Persistent Spatial-Line Estimation

For calibrated endpoint coordinates $p_{i}^{s},p_{i}^{e}\in R^{3}$ in view *i*, where $R^{3}$ denotes three-dimensional Euclidean space, the homogeneous image line is

(6) $$\ell_{i}=p_{i}^{s}\times p_{i}^{e}.$$

Let $P_{i}=\left[R_{i}|t_{i}\right]\in R^{3\times 4}$ be the world-to-camera projection matrix. The vector $\pi_{i}=P_{i}^{T}\ell_{i}\in R^{4}$ is the interpretation plane through the camera center and observed image line. Two nonparallel planes initialize the dual Plücker matrix [29]:

(7) $$L^{*}=\pi_{i}\pi_{j}^{T}-\pi_{j}\pi_{i}^{T}.$$

The landmark is optimized through a four-degree-of-freedom orthonormal representation. Thus, the finite endpoints remain image observations, while the persistent state represents the underlying infinite spatial line.

Let ${\tilde{u}}_{s}={\left[u_{s},v_{s},1\right]}^{T}$ and ${\tilde{u}}_{e}={\left[u_{e},v_{e},1\right]}^{T}$ be observed homogeneous endpoints, and let $\ell ={\left[a,b,c\right]}^{T}$ be the projection of a spatial line into that image. The geometric line residual is

(8) $$r_{L}=\left[\begin{array}{c} {\tilde{u}}_{s}^{T}\ell /\sqrt{a^{2}+b^{2}}\\ {\tilde{u}}_{e}^{T}\ell /\sqrt{a^{2}+b^{2}} \end{array}\right],$$

whose components are signed endpoint-to-projected-line distances. At keyframe *k*, the navigation state contains body position $p_{b_{k}}^{w}$, velocity $v_{b_{k}}^{w}$, orientation quaternion $q_{b_{k}}^{w}$, accelerometer bias $b_{a_{k}}$, and gyroscope bias $b_{g_{k}}$. IMU pre-integration follows the standard visual–inertial formulation [1,30].

Let X collect active navigation states, point landmarks, and line landmarks. Let B be consecutive keyframe pairs linked by IMU factors, C point observations, and L line observations. With marginalized prior $r_{0}$, information matrices $W_{\left(\cdot \right)}$, and robust loss ρ(·), the estimator minimizes

(9) $$\begin{array}{cc} \min_{X}\;\{ & \;{∥r_{0}∥}^{2}+\sum_{(i,j)\in B}{∥r_{B_{ij}}∥}_{W_{B}}^{2}\\ \; & +\sum_{q\in C}\rho \;\left({∥r_{P_{q}}∥}_{W_{P}}^{2}\right)+\sum_{h\in L}\rho \;\left({∥r_{L_{h}}∥}_{W_{L}}^{2}\right)\}. \end{array}$$

Here, $r_{B_{ij}}$, $r_{P_{q}}$, and $r_{L_{h}}$ are the IMU, point, and line residuals, respectively, and ${∥r∥}_{W}^{2}\;=\;r^{T}Wr$. Schur-complement marginalization retains information from states leaving the window in $r_{0}$.

### 3.5. Trajectory Evidence, Selection, and Integrity Audit

The EuRoC microaerial vehicle dataset provides synchronized camera and inertial data together with motion-reference trajectories across eleven sequences [31]. The evaluation uses the monocular stream and compares archived outputs from VINS-Mono, PL-VINS, and EDL-VINS. These systems were selected because complete primary trajectories and evo archives were retained for every sequence. They also provide a direct progression from the point-only parent estimator to its point–line extension and the evaluated EDLines/line-flow configuration. The selection criterion was the availability of complete archived trajectory outputs, not the availability of source code or runtime configurations. The evaluation is therefore a trajectory-level audit: it characterizes the accuracy and consistency of the archived outputs without requiring access to the implementations that produced them. Because the archived baselines were produced by their respective original pipelines and we did not re-execute them, we cannot confirm that VINS-Mono and PL-VINS were run under identical hardware or parameter settings; the protocol controls temporal support and alignment, but not the hardware or parameter provenance of the archived trajectories. The evidence package contains 33 primary estimated trajectories, 11 reference trajectories, and 33 evo result archives.

For each sequence and method, the trajectory stored at the root level of the corresponding archive was designated as the primary output before any metric was recomputed; therefore, the selection did not depend on the error obtained in this study. When two unique output-numbered files were available on MH_01 and MH_05, both were preserved for a separate descriptive analysis. Supplementary File records the source-to-package filename mapping and SHA-256 hash of every trajectory and evaluator archive.

Before numerical analysis, every estimated and reference trajectory was parsed and checked for finite values, strictly increasing timestamps, and quaternion norms near unity. Each evo archive contains a pose-error array and summary statistics. The audit independently recomputed the root-mean-square error (RMSE), mean, median, standard deviation, extrema, and sum of squared errors from each array and compared them with the stored statistics.

### 3.6. Protocol-Controlled Trajectory Analysis

The primary protocol establishes a shared 5 Hz grid over the temporal intersection of the reference and all three estimated trajectories for each sequence. Five hertz is the lowest nominal output rate among the retained primary trajectories. This grid frequency was selected after auditing the native output rates of all three evaluated systems across all eleven EuRoC sequences. VINS-Mono produces poses at a nominal 10 Hz, while PL-VINS and EDL-VINS produce poses at approximately 5–10 Hz with occasional internal gaps (up to 23.9 s on MH_01 for PL-VINS and 13.1 s for EDL-VINS). A common grid at the lowest native rate ensures that no method is evaluated at a frequency it cannot support, while avoiding the excessive interpolation that would result from using a much lower grid rate. The ground-truth reference at 200 Hz is downsampled to the same grid, ensuring that all four trajectories share identical temporal support. Sensitivity analyses on native timestamps confirm that the aggregate ranking is preserved under each method’s own output rate, demonstrating that the 5 Hz choice does not introduce a systematic bias. Position samples are linearly interpolated to this grid. Each estimate is then aligned independently to the reference by the rigid transformation $\left(R,t\right)\in \mathrm{SO}\left(3\right)\times R^{3}$, where SO(3) is the three-dimensional rotation group. The transformation is obtained by the Umeyama least-squares solution [32]:

(10) $$\left(R^{*},t^{*}\right)=\arg\min_{R\in \mathrm{SO}\left(3\right),t\in R^{3}}\sum_{k=1}^{K}{∥y_{k}-\left(R{\hat{y}}_{k}+t\right)∥}_{2}^{2},$$

where $y_{k}$ and ${\hat{y}}_{k}$ are the reference and estimated positions at common sample *k*, and *K* is the number of common samples. No scale correction is permitted in the primary protocol. The ATE RMSE is

(11) $${\mathrm{ATE}}_{\mathrm{RMSE}}=\sqrt{\frac{1}{K}\sum_{k=1}^{K}{∥y_{k}-\left(R^{*}{\hat{y}}_{k}+t^{*}\right)∥}_{2}^{2}}.$$

Temporal intersection controls the start and end of a comparison but does not prevent interpolation across an internal recording gap. Therefore, a gap-aware sensitivity analysis retains a grid time only when the reference and all three estimates either contain a sample at that time or have bracketing samples separated by no more than δ. We evaluate δ∈{0.3,0.5,1.0} s, spanning moderate to permissive multiples of the 0.2 s grid interval. Applying one shared validity mask at each threshold preserves identical support for all three methods.

ATE captures global trajectory agreement. To examine shorter-horizon behavior without relying on the archived quaternion ordering, the analysis also uses fixed-lag translational displacement error (DPE). Let ${\bar{y}}_{k}=R^{*}{\hat{y}}_{k}+t^{*}$, and let *h* be a lag in grid samples. The displacement error is

(12) $$d_{k}^{\left(h\right)}={∥\left({\bar{y}}_{k+h}-{\bar{y}}_{k}\right)-\left(y_{k+h}-y_{k}\right)∥}_{2},$$

and its RMSE is reported for lags of 1 s and 5 s. The 1 s lag captures short-horizon motion, whereas the 5 s lag shows how translational disagreement accumulates over a longer local interval. DPE is not presented as a substitute for full SE(3) relative pose error.

In addition to the gap-aware analysis, two alignment and support checks accompany the primary protocol. First, the same 5 Hz samples are aligned by Sim(3), allowing a uniform scale factor. Second, the original evo archives are summarized on each method’s native timestamp support with their recorded Sim(3) alignment. The sequence is the paired unit for method differences. A deterministic paired bootstrap with 100,000 resamples and seed 20260717 estimates 95% percentile intervals for the mean ATE difference. Finally, the two unique stored outputs per method on MH_01 and MH_05 are evaluated separately with SE(3) alignment. This last comparison describes the variation present in the archive; it does not change the sequence-level unit used for inference.

## 4. Results

The integrity audit established the numerical basis of the comparison before any ranking was computed. All 44 trajectory files passed the finite-value and timestamp checks, and their quaternion norms remained near unity. Statistics recomputed from the 33 evo error arrays matched the archived values to the stored numerical precision. The audit also showed that an earlier unpublished summary table had placed the archived EDL-VINS standard deviation in the V2_01 RMSE cell. The present analysis uses the recomputed RMSE, so this correction precedes every aggregate and paired comparison.

The results that follow build on this audit as a causal chain: from the retained detector-stage record and qualitative visual support to the primary common-support accuracy, its sensitivity to alignment and timestamp support, shorter-horizon and rotational behavior, and finally, the difficult, exception, and archived-output cases that bound the confidence placed in the ranking.

### 4.1. Detector-Stage Record and Visual Support

The retained detector record compares LSD with the conditioned EDLines stage at three resolutions. Timing was recorded on an NVIDIA Jetson Orin Nano (NVIDIA Corporation, Santa Clara, CA, USA) with 8 GB RAM. Table 3 reports the candidate count before the 100-line back-end budget and detector-stage runtime. The EDLines-based stage returns a similar number of candidates with lower recorded extraction time at each resolution. These measurements concern line extraction only; line flow and optimization are evaluated through trajectory behavior rather than folded into the timing claim.

**Figure 2:**
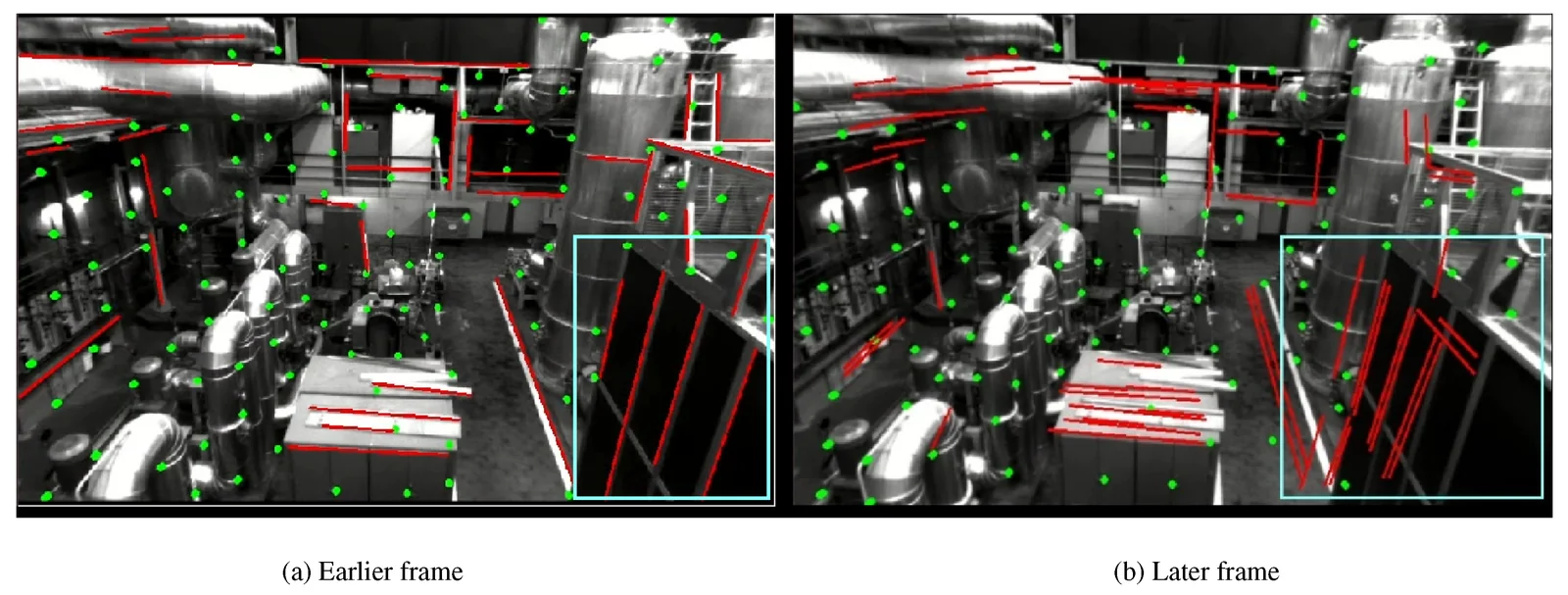
Point and line observations in two consecutive images: (**a**) earlier frame and (**b**) later frame. Red segments denote detected line support, green points denote tracked corner features, and cyan rectangles mark structural regions for visual inspection.

Figure 2 provides the corresponding qualitative context. Structural boundaries remain represented across consecutive images, while point observations concentrate in textured regions. The figure provides qualitative evidence of complementary visual support, and the trajectory outcomes provide the quantitative system-level assessment.

The timing data in Table 3 are limited. Only the detector stage was instrumented, so end-to-end pipeline runtime (feature tracking, IMU preintegration, optimization, and marginalization) is not reported; each resolution was recorded as a single measurement without repetitions, so no variance or confidence interval is available; and the record contains no memory-usage or line-tracking-quality metrics. Accordingly, no end-to-end runtime or real-time-operation claim is made in this work: the measurements characterize only the line-extraction stage, and system-level behavior is assessed through the trajectory analyses below. A comprehensive hardware benchmark—including repeated end-to-end CPU/GPU timing, peak memory, and line-track survival rate—is planned on the same Jetson Orin Nano (8 GB) platform as future work.

### 4.2. Common-Support EuRoC Accuracy

Table 4 reports the primary 5 Hz common-support, SE(3)-aligned ATE. EDL-VINS has the lowest arithmetic mean and the lowest sequence RMSE in eight sequences. VINS-Mono is lowest on V1_01, and PL-VINS is lowest on V1_03 and V2_01. Relative to each comparator separately, EDL-VINS is lower on nine sequences and higher on two. Thus, the aggregate pattern favors EDL-VINS without implying uniform dominance.

**Figure 3:**
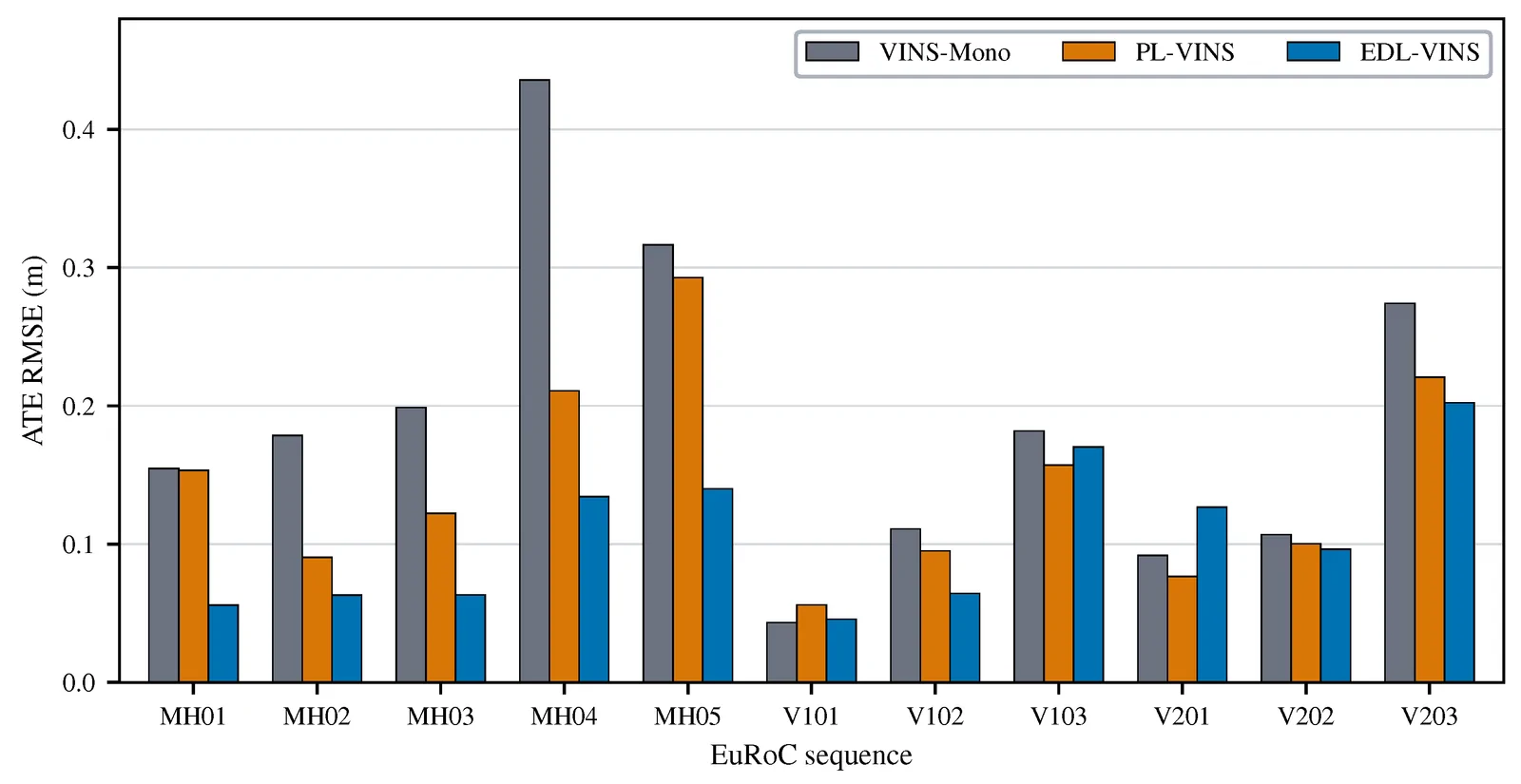
Common-support EuRoC ATE RMSE under SE(3) alignment. The bars are generated directly from Table 4. Sequence abbreviations omit underscores for legibility.

The mean paired RMSE reduction is 0.085 m relative to VINS-Mono and 0.038 m relative to PL-VINS. The corresponding sequence-level paired-bootstrap 95% intervals are [0.034, 0.143] m and [0.007, 0.070] m. These intervals quantify variation across the eleven sequence blocks. Figure 3 shows the complete cross-sequence pattern, including the V2_01 reversal that was obscured by the historical transcription error.

### 4.3. Alignment and Timestamp-Support Sensitivity

Table 5 compares the primary protocol with three sensitivity analyses. Allowing scale correction lowers all three aggregate errors, but it does not change the number of sequence wins. Native timestamp support produces nearly the same aggregate ordering as the 5 Hz Sim(3) grid. Native-support SE(3) alignment, included in response to the question of whether 5 Hz resampling introduces bias, leaves the PL-VINS and EDL-VINS means unchanged at 0.143 m and 0.106 m and raises the VINS-Mono mean from 0.190 m to 0.192 m, a difference of 0.002 m or about 1%; because the lowest-sequence counts are identical under both protocols, the primary metric-scale conclusion is insensitive to the resampling choice. The consistency indicates that the aggregate ranking is not created by interpolation or one particular support rule. The difference between SE(3) and Sim(3) remains scientifically relevant, however, because it reveals a scale error that the corrected protocol removes.

The largest EDL-VINS scale adjustment occurs on V2_01, where the common-grid Sim(3) scale is 1.043. Its RMSE decreases from 0.127 m under SE(3) to 0.083 m under Sim(3), yet PL-VINS remains lower. This sequence therefore exposes a genuine scale-sensitive failure case rather than a rounding-level exception.

Temporal intersection alone does not remove internal recording gaps. Table 6 therefore repeats the primary SE(3) analysis after excluding every grid time that would require any input trajectory to interpolate across a gap larger than δ. The retained grid fraction ranges from 77.7% to 100.0% at the strictest threshold and increases as the threshold is relaxed. Across all three thresholds, the sequence-win counts remain 1, 2, and 8 for VINS-Mono, PL-VINS, and EDL-VINS, respectively, while the aggregate errors change only slightly. Thus, the reported ordering is not produced by interpolation across long internal gaps.

Per-sequence valid-grid fractions and method-specific errors for every value of δ are reported in Supplementary File in results/gap_aware_sensitivity_metrics.csv.

### 4.4. Fixed-Lag Translational Behavior

Table 7 separates global trajectory agreement from shorter-horizon translation. EDL-VINS has the lowest mean DPE at both lags, but it is lowest on only six sequences. The 1 s mean is close to VINS-Mono, whereas the 5 s lag shows a larger aggregate separation. This result narrows the interpretation of Table 4: EDL-VINS exhibits the strongest aggregate global ATE in the archive, but its local displacement behavior is more sequence-dependent.

### 4.5. Rotational Error Analysis

The preceding analysis concerns translational displacement; we now turn to rotational accuracy, which is not reflected in position-error metrics. Table 8 reports rotational accuracy computed from the quaternion components of the same 5 Hz common-support trajectories used for the translational analysis.

The rotational protocol is specified as follows. Orientations use Hamilton quaternions, stored scalar-last, $\left(q_{x},q_{y},q_{z},q_{w}\right)$, in the released trajectory files. For each sequence, the reference and estimated orientation trajectories are resampled onto the shared 5 Hz grid of Section 3.6 by normalized linear quaternion interpolation (nlerp) with hemisphere sign correction. The Umeyama rotation $R^{*}$ obtained from the position alignment of Section 3.6 is then applied to the estimated orientations, ${\hat{q}}_{k}\leftarrow R^{*}⊗{\hat{q}}_{k}$, and a first-pose registration $q_{\mathrm{reg}}=q_{1}⊗{\left(R^{*}⊗{\hat{q}}_{1}\right)}^{-1}$ removes the residual orientation offset at the first grid sample, so that every method shares the same rotational reference by construction. The error quaternion at grid sample *k* is $q_{k}^{e}=q_{k}^{-1}⊗{\hat{q}}_{k}$, with scalar part $w_{k}^{e}$ and vector part $v_{k}^{e}$. The rotational ATE is the RMSE of the error angle $\theta_{k}=2\;\mathrm{atan}2(∥v_{k}^{e}∥,|w_{k}^{e}\left|\right)$ over the grid, and the per-axis roll, pitch, and yaw RMSE values are obtained from the ZYX Euler decomposition of $q_{k}^{e}$. The rotational RPE at lag *h* replaces absolute orientations with relative rotations $q_{k,h}^{\Delta }=q_{k}^{-1}⊗q_{k+h}$ and reports the RMSE of the angle of ${\left(q_{k,h}^{\Delta }\right)}^{-1}⊗{\hat{q}}_{k,h}^{\Delta }$ for h∈{1,5} s; because the global alignment and first-pose registration cancel in the relative rotations, the RPE values depend only on the short-horizon behavior of each estimate. The values in Table 8 are the mean of the per-sequence RMSE values over the eleven sequences, and the per-sequence values together with the analysis script are released in Supplementary File (analysis/rotation_metrics.py, results/rotation_per_sequence_metrics.csv, results/rotation_summary.csv).

EDL-VINS has the lowest rotation ATE (3.763°) and the lowest aggregate roll and yaw RMSE, consistent with its translational advantage. However, the short-horizon rotational RPE at the 1 s lag shows the opposite ordering: VINS-Mono has the lowest value (0.389°), while EDL-VINS is highest (0.535°). This echoes the translational DPE result in Table 7, where the EDL-VINS margin over VINS-Mono narrows to 0.002 m at the 1 s lag before widening to 0.013 m at the 5 s lag. The rotation analysis therefore confirms that EDL-VINS excels in global accuracy but does not uniformly dominate short-horizon precision, a finding that further bounds the interpretation of the aggregate ATE advantage.

### 4.6. Difficult and Exception Sequences

**Figure 4:**
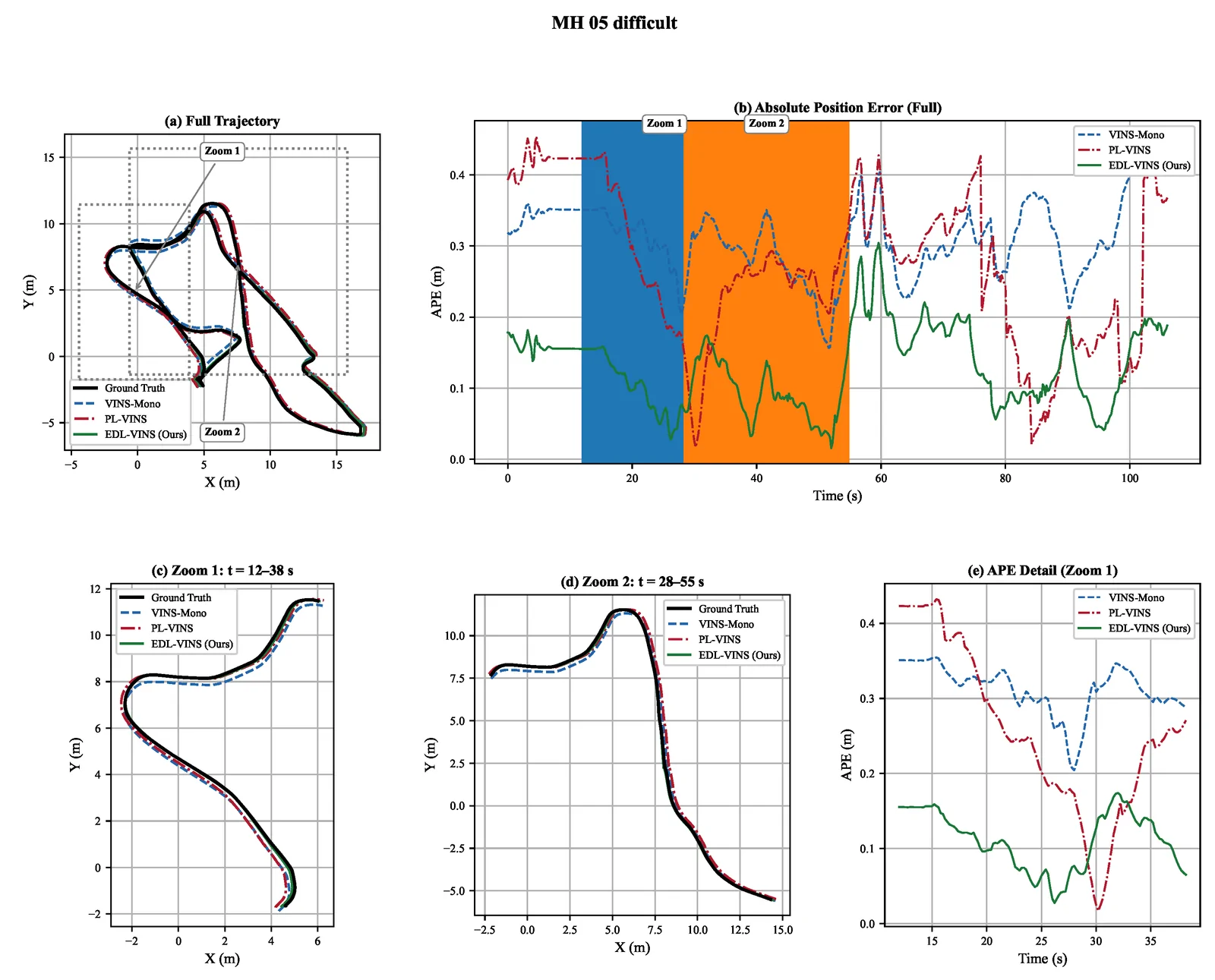
MH_05_difficult under the primary protocol: (**a**) full aligned planar trajectories, (**b**) absolute position error over elapsed time, (**c**) zoomed start-segment trajectories, (**d**) zoomed middle-segment trajectories, and (**e**) zoomed first-30-s position error. Zoomed subplots are provided to resolve trajectory overlap in dense regions. Subplots use the same common 5 Hz samples as Table 9.

MH_05 is examined because it combines rapid motion, illumination variation, and limited texture. Table 9 and Figure 4 are generated from the same common-grid SE(3) analysis as the main benchmark. EDL-VINS has the lowest mean, median, and RMSE on this sequence. Its error remains below the two comparators for most of the retained interval, although all methods show localized increases around high-curvature motion.

**Figure 5:**
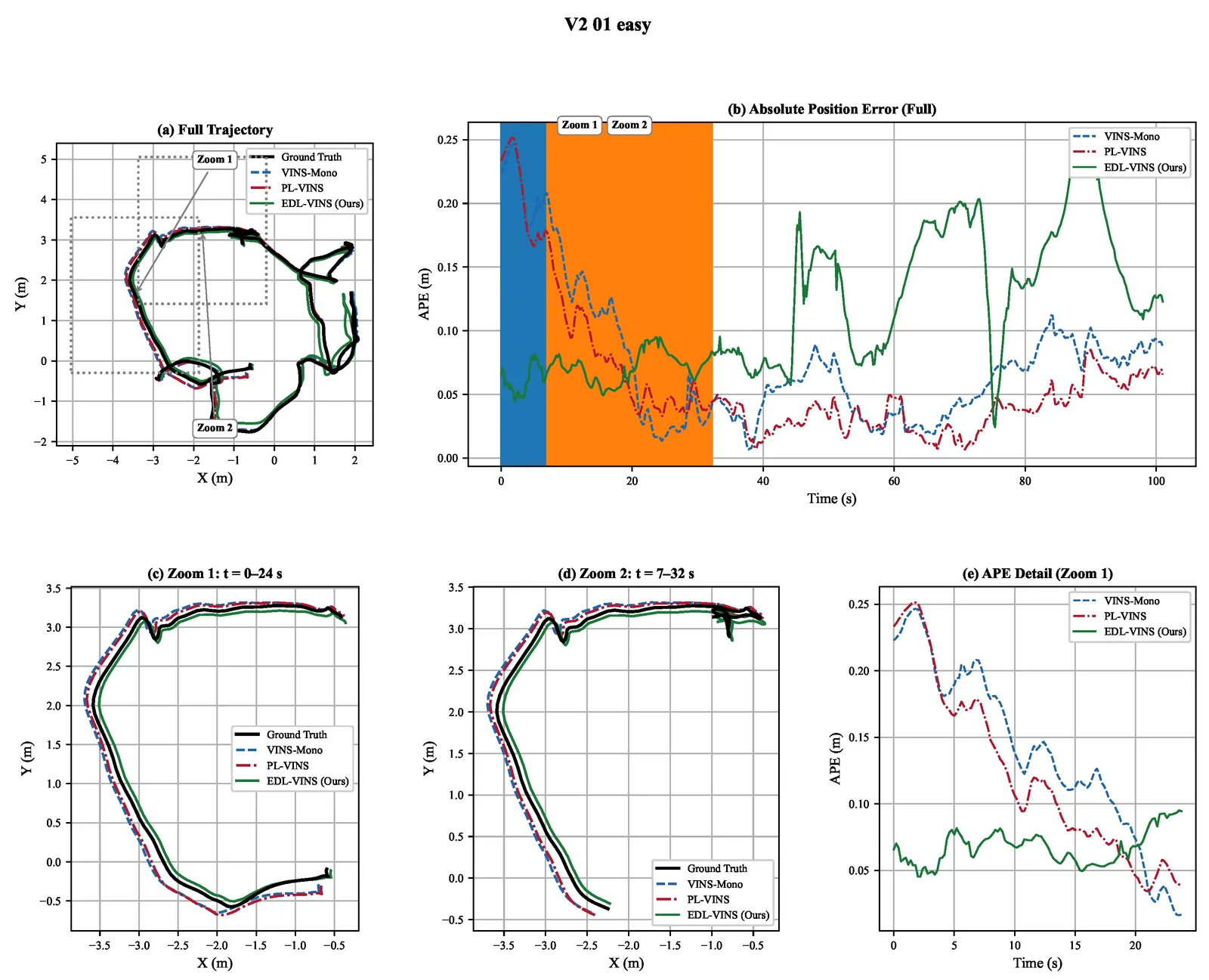
V2_01_easy under the primary protocol: (**a**) full aligned planar trajectories, (**b**) absolute position error over elapsed time, (**c**) zoomed start-segment trajectories, (**d**) zoomed middle-segment trajectories, and (**e**) zoomed first-30-s position error. Zoomed subplots resolve trajectory overlap in dense regions. This sequence is an EDL-VINS exception and has the largest EDL-VINS scale correction in the sensitivity analysis.

V2_01 provides the complementary exception. Figure 5 shows that EDL-VINS develops a larger position error through the middle and later portions of the sequence, while the two comparators remain closer to the reference. Reporting this case beside MH_05 prevents the detailed analysis from selecting only a favorable sequence and identifies scale preservation as a practical evaluation concern.

### 4.7. Selected Archived-Output Variation

The archive contains two unique outputs per method for MH_01 and MH_05. Table 10 summarizes independently aligned RMSE values on each output’s native temporal support. The two EDL-VINS RMSE values differ by 0.00124 m on MH_01 and 0.00006 m on MH_05. By comparison, the PL-VINS difference is 0.00104 m on MH_01 and 0.03862 m on MH_05. Expressed relative to the corresponding mean, the spread between the two archived EDL-VINS outputs is 2.1% on MH_01 and below 0.1% on MH_05 (coefficient of variation 0.015 and 0.0003), whereas the PL-VINS spread is 0.7% on MH_01 but reaches 15.0% on MH_05 (coefficient of variation 0.005 and 0.106). PL-VINS is therefore marginally the more consistent of the two on MH_01 values, while its MH_05 spread is the largest archived-output variation observed. These figures describe only differences between distinct archived files, not run-to-run repeatability: the retained outputs were stored as separate numbered files within each archive rather than generated by re-executing the system, so true run-to-run uncertainty remains unmeasured and is not captured by the sequence-level paired bootstrap. Because the primary ranking in Table 4 rests on a single retained run per method per sequence, this archived-output variation bounds the confidence placed in that ranking—a method whose outputs vary at the PL-VINS level on MH_05 could change its per-sequence win count under re-execution. The paired-bootstrap intervals quantify alignment sensitivity, not run-to-run certainty, and should not be read as evidence of high repeatability; the corresponding limitation is taken up in Section 5. The unverifiable hardware and parameter provenance of the archived baselines (Section 3.5) further bounds how these spreads should be read. Because VINS-Mono and PL-VINS were executed by their original pipelines under settings that could not be confirmed, part of their archived-output spread—most visibly the 15.0% PL-VINS spread on MH_05—may reflect differences in execution hardware or parameter configuration rather than algorithmic randomness alone. Table 10 therefore quantifies the variation present in the archived evidence under its unknown provenance; it is not a controlled measurement of each method’s intrinsic run-to-run variability.

## 5. Discussion

The protocol-controlled results answer the central question affirmatively at the aggregate level: the lower mean ATE of the evaluated EDL-VINS configuration persists when all methods share the same timestamps and metric scale is preserved. The same ordering also remains after long interpolation gaps are removed and under the scale-corrected and native-support checks. At the same time, the V1_01, V1_03, and V2_01 rankings and the fixed-lag analysis show that no method dominates every motion regime or temporal scale.

The system design provides a plausible explanation for the lower aggregate ATE. Conditioned EDLines supplies compact structural support, line flow avoids descriptor computation for every active segment, and multi-view Plücker landmarks transfer accepted image-line tracks into endpoint-independent geometric factors. This division is important because detected endpoints delimit visible support and can move along the same physical edge, whereas the spatial-line state represents persistent scene geometry. The detector record and trajectory analysis support the complete configuration at their respective stages, but they should not be combined into an unmeasured end-to-end runtime claim.

Relative to the closest work, EDL-VINS is best understood as an evaluated integration in the EPLF-VINS/PL-VINS design family. EPLF-VINS introduced EDLines conditioning, line optical flow, and endpoint-independent line measurement [6]; EPL-VINS further combined optimized EDLines processing and line flow with an adaptive residual [25]; and PL-VINS supplied the VINS-derived point–line estimator architecture [4]. The present study contributes a protocol-controlled characterization of one retained configuration, including correction of a historical metric transcription, scale and interpolation-gap sensitivity, common-support analysis, local displacement behavior, and released evaluation artifacts. The resulting finding combines robustness with a clear boundary: the aggregate ordering survives stricter support and alignment controls, but it does not extend uniformly across sequences or temporal scales. This is an empirical and reproducibility-centered contribution rather than a claim that the inherited front-end operations are new. While the individual algorithmic elements are inherited from EPLF-VINS and PL-VINS, the evaluated configuration has not previously been characterized under a protocol that controls for temporal support, alignment, and scale treatment. The contribution is therefore the evaluation methodology itself and the auditable evidence it produces, rather than a claim of algorithmic novelty.

The comparison also illustrates why evaluation protocol is part of the scientific result. On V2_01, scale correction reduces the EDL-VINS error substantially but does not reverse the corrected ranking. A native-support table containing the previous transcription would instead report an apparent win. Common support, explicit SE(3) alignment, and direct recomputation from pose-error arrays prevent those two effects from being conflated with estimator quality. The same principles apply beyond the three implementations studied here.

The conclusions are bounded by the archived evidence. They apply to translational and rotational accuracy on EuRoC for three retained implementations, but do not establish behavior in dynamic or outdoor settings, nor a current state-of-the-art ranking. Because versioned source and configuration snapshots, controlled module substitutions, independent repeated runs, and end-to-end resource logs were not retained, implementation-level reproduction, causal attribution, repeatability, and end-to-end efficiency remain open. This limitation means that the trajectory-level results in Table 4, Table 5, Table 6, Table 7, Table 8, Table 9 and Table 10 are auditable at the data level (all trajectory files, evaluation arrays, and analysis code are released), but the implementation-level pipeline that generated those trajectories cannot be independently re-executed. The released evidence package therefore supports verification of the evaluation protocol and its conclusions, while acknowledging that implementation-level reproduction remains an open task.

Three additional limitations bound the scope of the conclusions. First, the evaluation does not include a controlled ablation isolating the contributions of EDLines conditioning, line flow, and Plücker-line factors; the reported ATE gains therefore characterize the complete configuration rather than individual components. An archived variant-B configuration (using algebraic instead of normalized line residuals) was evaluated on MH_01 and produced trajectory divergence, providing indirect evidence that the normalized residual formulation is essential, but this observation does not constitute a systematic ablation. A systematic ablation would require independent module substitutions (e.g., EDLines conditioning versus LSD, line flow versus descriptor matching, normalized versus algebraic residuals) executed within the complete pipeline, which the archived build does not retain; component-level attribution therefore lies outside the present scope. Second, the comparison is limited to VINS-Mono and PL-VINS as directly comparable baselines. Although EDL-VINS belongs to the EPLF-VINS/PL-VINS design family—with EPLF-VINS and EPL-VINS being its most direct siblings in terms of inherited line-flow and Plücker-line design—the two parent systems enter the benchmark differently. EPL-VINS outputs were not retained in the evidence package, so EPL-VINS appears only as a literature-reported reference column in Table 4 under the original authors’ protocols. EPLF-VINS trajectories, by contrast, were retained; in this revision, they were re-executed with the authors’ released code under the present 5 Hz common-grid SE(3) protocol without loop closure on the three retained sequences, appearing as the directly comparable EPLF Re-Run column in Table 4. Identical-protocol re-execution of the remaining EPL-VINS sibling remains an explicit next step enabled by the released evidence framework. Beyond the siblings, additional external baselines such as OKVIS, ORB-SLAM3, or more recent point–line systems would also strengthen the evaluation. Third, validation is restricted to the EuRoC dataset; no outdoor or dynamic-environment datasets were evaluated, and a short corridor sequence captured on the platform lacks surveyed ground truth for quantitative validation. These limitations restrict generalization claims to the EuRoC benchmark under the evaluated configurations. Supplementary File nevertheless permits direct audit and reproduction of the trajectory-level comparisons.

## 6. Conclusions

This study evaluated an EDLines and line-flow point–line visual–inertial system under a protocol that preserves metric scale and assigns identical temporal support to all compared trajectories. EDL-VINS retains the lowest aggregate EuRoC ATE and rotational error under this protocol, and the ordering remains stable when scale treatment, timestamp support, and interpolation gaps are varied. The advantage is nevertheless sequence- and timescale-dependent, as demonstrated by the fixed-lag results and the V2_01 exception. More broadly, trajectory claims for visual–inertial localization are most credible when raw outputs, temporal support, alignment choices, correction history, and analysis code are exposed. The released evidence package provides this audit trail for the evaluated configuration.

In practical terms, the evaluated configuration targets robots operating in structured but weakly textured indoor environments—microaerial vehicles inspecting corridors, tunnels, or industrial halls, as well as ground robots navigating warehouses or similar facilities—where line structure remains observable when point features degrade. Two properties of the evaluated design are directly relevant to such deployments: the conditioned EDLines stage reduces detector-stage extraction time by about an order of magnitude relative to LSD at each evaluated resolution on the embedded platform (Table 3), easing the computational budget of resource-limited onboard processing, and line flow associates segments without per-frame descriptor computation for every active segment. Beyond the specific configuration, the released protocol benefits practitioners who must compare visual–inertial systems retrospectively: the common-support grid, explicit SE(3) alignment, gap-aware masking, and released analysis code permit archived trajectories to be re-audited without access to the original implementations. At the same time, the conclusions of this study are strictly limited to the retained trajectory-level evaluation on EuRoC: they do not extend to component-level attribution, which requires controlled ablations; to run-to-run repeatability, which requires independent repeated executions; to end-to-end runtime, which was not measured; or to other datasets and environments.

## Figures and Tables

**Table 1:** Representative point–line visual–inertial systems and their line-processing interfaces.

System	Line Front End	Temporal Association	Estimator-Side Line Role
PL-VIO [3]	LSD and LBD	Descriptor matching with geometric rejection	Plücker/orthonormal landmarks in tightly coupled VIO
PL-VINS [4]	Modified LSD-family detector	Descriptor-based segment matching	VINS-Mono-derived point–line estimator
PLI-VINS [8]	Adaptive extraction and merging	LBD and geometric checks	Point–line–inertial sliding window
LRPL-VIO [9]	Selected endpoint and midpoint samples	IMU-predicted photometric tracking	Multi-view filter update
EPLF-VINS [6]	EDLines fusion, spatial control, and adaptive extraction	Pyramidal line optical flow	Endpoint-independent geometric line measurement
EPL-VINS [25]	Optimized EDLines processing	Line flow with geometric validation	Adaptive point–line estimator residual
EDL-VINS	Conditioned EDLines candidate set	Pyramidal line optical flow	Multi-view Plücker landmarks and geometric line factors

Abbreviations: LSD, Line Segment Detector; LBD, Line Band Descriptor; VIO, visual–inertial odometry; IMU, inertial measurement unit.

**Table 2:** Numerical settings preserved for the evaluated EDL-VINS configuration.

Setting	Recorded Value
Fragment-fusion angle threshold	$5^{\circ }$
Fragment endpoint-proximity threshold	10 pixels
Minimum accepted segment length	30 pixels
Adaptive detection trigger	Fewer than 40 active segments
Low-texture line recovery (code-verified)	active line count <40 lowers the EDL+ gradient threshold and re-runs the EDLines detector to recover cues; segments <30 px discarded; per-frame segment budget 100
EDL+ adaptive gradient threshold (paper, Section 3)	EDLines-based front end (Akinlar & Topal, 2011 [5]); gradient threshold lowered under low-texture trigger; exact baseline recorded in the implementation record
Line budget passed to the back end	100 segments per frame
Sliding-window size	10 keyframes
Line-flow tracking window	21×21 pixels
Line-flow pyramid levels	3
Point-feature tracking window	21×21 pixels
Point-feature pyramid levels	3
Reverse-check displacement threshold	0.5 pixels

**Table 3:** Retained line-extraction record on an NVIDIA Jetson Orin Nano with 8 GB RAM. Runtime is detector-stage time in milliseconds; smaller values indicate lower detector-stage processing time.

Resolution	Method	Candidates	Time (ms)
256×256	LSD	220	30.86
	Conditioned EDLines	198	3.36
480×320	LSD	399	73.40
	Conditioned EDLines	354	7.86
512×512	LSD	721	100.54
	Conditioned EDLines	667	12.41

**Table 4:** ATE RMSE in meters on a common 5 Hz time grid with SE(3) alignment and no scale correction. Bold values are the lowest among the three evaluated methods in each row. Columns marked ^a^ report literature-published point–line and stereo-inertial ATE (EPLF-VINS, EPL-VINS, ORB-SLAM3, OKVIS2) under the original authors’ protocols and are provided for context only; they are not directly comparable to the present 5 Hz common-grid SE(3) evaluation. An additional column, EPLF-VINS (Re-Run), reports EPLF-VINS re-executed with the authors’ released code under the present 5 Hz common-grid SE(3) protocol without loop closure (default configuration) on the three sequences with retained trajectories (V1_02, V2_02, V2_03); it is directly comparable to the three evaluated methods and complements the literature-reported reference.

Sequence	VINS-Mono	PL-VINS	EDL-VINS	EPLF ^a^	EPLF Re-Run	EPL ^a^	ORB-SLAM3 ^a^	OKVIS2 ^a^
MH_01_easy	0.155	0.153	**0.056**	0.039	—	0.068	0.036	0.027
MH_02_easy	0.179	0.090	**0.063**	0.072	—	0.071	0.023	0.023
MH_03_medium	0.199	0.122	**0.063**	0.047	—	0.063	0.028	0.028
MH_04_difficult	0.436	0.211	**0.134**	0.125	—	0.142	0.066	0.066
MH_05_difficult	0.317	0.293	**0.140**	0.089	—	0.112	0.082	0.068
V1_01_easy	**0.043**	0.056	0.046	0.046	—	—	0.035	0.035
V1_02_medium	0.111	0.095	**0.064**	0.034	0.169	0.047	0.013	0.013
V1_03_difficult	0.182	**0.157**	0.170	0.123	—	—	0.019	0.019
V2_01_easy	0.092	**0.077**	0.127	0.096	—	—	0.023	0.023
V2_02_medium	0.107	0.100	**0.096**	0.058	0.147	0.081	0.015	0.015
V2_03_difficult	0.274	0.221	**0.202**	0.149	0.500	—	0.024	0.020
Arithmetic mean	0.190	0.143	**0.106**	0.080	0.272 ^‡^	0.083 ^†^	0.033	0.031

^a^ Literature-reported ATE RMSE (m) under each system’s original evaluation protocol and therefore not directly comparable to the present 5 Hz common-grid SE(3) evaluation. EPLF-VINS: Xu et al. [6]; EPL-VINS: Jin et al. [25]; ORB-SLAM3: Campos et al. [33]; OKVIS2: Leutenegger [34]. ^†^ EPL-VINS mean computed over the seven sequences reported in the literature (V1_01, V1_03, V2_01, and V2_03 were not reported). ^‡^ EPLF-VINS (Re-Run) re-executed with the authors’ released code under the present 5 Hz common-grid SE(3) protocol without loop closure (default configuration) on the three sequences with retained trajectories (V1_02, V2_02, V2_03), spanning medium-to-difficult regimes; it is directly comparable to the evaluated methods and remains higher than the literature-reported EPLF-VINS because the published results use the authors’ tuned configuration. A dash (—) indicates that no trajectory could be obtained for that sequence under the common protocol; such entries are excluded from the comparison rather than imputed.

**Table 5:** Protocol sensitivity of sequence-level ATE RMSE. Means are arithmetic averages over eleven sequence RMSE values.

Protocol	Method	Mean RMSE (m)	Lowest-Sequence Count
Common 5 Hz, SE(3)	VINS-Mono	0.190	1
	PL-VINS	0.143	2
	EDL-VINS	**0.106**	**8**
Common 5 Hz, Sim(3)	VINS-Mono	0.184	1
	PL-VINS	0.134	2
	EDL-VINS	**0.097**	**8**
Native support, archived Sim(3)	VINS-Mono	0.186	1
	PL-VINS	0.134	2
	EDL-VINS	**0.097**	**8**
Native support, SE(3)	VINS-Mono	0.192	1
	PL-VINS	0.143	2
	EDL-VINS	**0.106**	**8**

**Table 6:** Gap-aware sensitivity under common 5 Hz support and SE(3) alignment. Valid-grid coverage is the minimum/mean/maximum percentage across eleven sequences. Bold values are the lowest mean RMSE in each row.

δ (s)	Valid-Grid Coverage (%)	VINS-Mono (m)	PL-VINS (m)	EDL-VINS (m)
0.3	77.7/89.6/100.0	0.188	0.141	**0.106**
0.5	85.1/94.3/100.0	0.190	0.142	**0.106**
1.0	86.5/95.7/100.0	0.190	0.143	**0.106**

**Table 7:** Sequence-averaged fixed-lag displacement-error RMSE on the common 5 Hz grid. The lowest-sequence count records the number of sequences on which each method has the smallest DPE. Bold values indicate the lowest mean DPE RMSE at each lag.

	1 s Lag		5 s Lag	
Method	Mean RMSE (m)	Lowest Count	Mean RMSE (m)	Lowest Count
VINS-Mono	0.039	1	0.106	2
PL-VINS	0.048	4	0.116	3
EDL-VINS	**0.037**	**6**	**0.093**	**6**

**Table 8:** Rotational error summary (degrees) on the common 5 Hz grid. Roll, pitch, and yaw are per-axis RMSE; ATE is the overall rotation error; RPE is the fixed-lag rotational relative-pose error. Bold values are the lowest in each column.

Method	Roll	Pitch	Yaw	ATE	RPE-1 s	RPE-5 s
VINS-Mono	3.374	0.937	1.281	3.858	**0.389**	**1.003**
PL-VINS	3.362	**0.878**	1.294	3.838	0.525	1.154
EDL-VINS	**3.294**	0.948	**1.275**	**3.763**	0.535	1.101

**Table 9:** Common-grid absolute position-error statistics on MH_05_difficult, in meters. Bold values indicate the lowest value in each row.

Statistic	VINS-Mono	PL-VINS	EDL-VINS
Mean	0.312	0.272	**0.129**
Median	0.314	0.270	**0.129**
RMSE	0.317	0.293	**0.140**
Standard deviation	**0.054**	0.108	0.055

**Table 10:** Archived-output ATE under SE(3) alignment. SD is the sample standard deviation across the two unique stored outputs for that method and sequence; range is the maximum RMSE minus the minimum RMSE.

Sequence	Method	Outputs	Mean RMSE (m)	SD (m)	Range (m)
MH_01_easy	VINS-Mono	2	0.15505	0.00003	0.00004
	PL-VINS	2	0.14803	0.00074	0.00104
	EDL-VINS	2	0.05989	0.00087	0.00124
MH_05_difficult	VINS-Mono	2	0.31709	<0.00001	<0.00001
	PL-VINS	2	0.25730	0.02731	0.03862
	EDL-VINS	2	0.13319	0.00004	0.00006

## Data Availability

The EuRoC microaerial vehicle dataset is publicly available [31]. All estimated trajectories, reference trajectories used by the analysis, archived evaluator arrays, integrity hashes, derived tables, and scripts required to reproduce the primary and sensitivity results in this article are provided in Supplementary File.

## Supplementary Materials

The following supporting information can be downloaded at https://www.mdpi.com/article/10.3390/s26175508/s1.

## Abbreviations

The following abbreviations are used in this manuscript:

ATE	Absolute trajectory error
DPE	Displacement error
EDL-VINS	EDLines visual–inertial navigation system
IMU	Inertial measurement unit
LBD	Line Band Descriptor
LSD	Line Segment Detector
RMSE	Root-mean-square error
SLAM	Simultaneous localization and mapping
VIO	Visual–inertial odometry
